# The Image Clarity Paradox: Higher CZT SPECT Contrast Does Not Always Translate to Diagnostic Accuracy for Alzheimer’s Disease

**DOI:** 10.3390/tomography11060061

**Published:** 2025-05-25

**Authors:** Kiyotaka Nemoto, Bryan J. Mathis, Akemi Iwasaka, Kenjiro Nakayama, Tomohiro Kaneta, Tetsuaki Arai

**Affiliations:** 1 Department of Psychiatry, Institute of Clinical Medicine, University of Tsukuba, Tsukuba 305-8575, Japan; 2 Department of Cardiovascular Surgery, Institute of Clinical Medicine, University of Tsukuba, Tsukuba 305-8575, Japan; 3 Department of Radiology, University of Tsukuba Hospital, Tsukuba 305-8576, Japan; 4 Doctoral Program in Medical Sciences, Graduate School of Comprehensive Human Sciences, University of Tsukuba, Tsukuba 305-8574, Japan; 5 Department of Diagnostic Image Analysis, Graduate School of Medicine, Tohoku University, Sendai 980-8575, Japan

**Keywords:** single-photon emission computed tomography (SPECT), cerebral perfusion SPECT, cadmium–zinc–telluride (CZT) detector, sodium iodide (NaI) scintillation detector

## Abstract

**Background**: Recent advances in single-photon emission computed tomography (SPECT) technology, particularly cadmium–zinc–telluride (CZT) detectors, have improved spatial resolution and contrast in cerebral blood flow imaging. This study aimed to investigate whether these improvements translate to enhanced diagnostic accuracy for Alzheimer’s disease (AD). **Methods**: We compared conventional SPECT (eCAM) with CZT SPECT in 29 patients (mean age 60.9 ± 17.6 years, 69% female) with suspected neurodegenerative diseases. **Results**: Gray matter/white matter contrast was significantly higher in CZT SPECT compared to eCAM (1.615 ± 0.096 vs. 1.458 ± 0.068, *p* < 0.001). However, diagnostic accuracy for AD did not improve with CZT SPECT. For the participating psychiatrist, sensitivity decreased from 0.750 (eCAM) to 0.625 (CZT), while for the radiologist, specificity dropped from 0.571 (eCAM) to 0.429 (CZT). Overall accuracy slightly decreased for both readers. **Conclusions**: These findings suggest that while CZT SPECT offers superior image quality, it may not immediately translate to improved diagnostic accuracy for AD. The study highlights the importance of specialized training for clinicians in interpreting higher-resolution CZT SPECT images to fully leverage their potential in neurodegenerative disease diagnosis. Future research should focus on developing standardized training protocols and larger, multi-center studies to validate these findings.

## 1. Introduction

Neurodegenerative conditions, such as Alzheimer’s disease, are maladies of cerebral metabolism and blood flow. The advent of single-photon emission computed tomography (SPECT) allows for imaging of these biomarkers by compositing anatomical data with flow and glucose metabolism. However, typical limitations on the spatial resolution of anatomical landmarks (around 10 mm at best) may reduce visual rating and inter-reader reliability with respect to early detection of disease. Statistical transforms of SPECT data, such as the easy Z-score imaging system (eZIS) [1], are thus used to increase the accuracy of differential diagnosis. However, in spite of these methods, highly precise differential diagnosis at early stages of disease remains arduous because of the low anatomical resolution.

**Figure 1:**
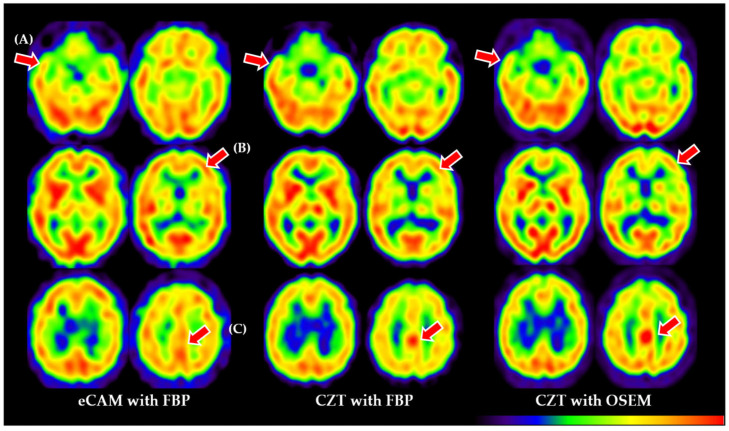
Representative brain scans showing key differences between eCAM with FBP (**left**), CZT with FBP (**center**), and CZT with OSEM (**right**). Red arrows indicate (**A**) improved temporal lobe definition in CZT images; (**B**) enhanced gray/white matter contrast, with sharper boundaries visible in CZT images; (**C**) different visualization of the posterior cingulate gyrus, a critical region for Alzheimer’s disease diagnosis. Note the progressively improved image clarity from left to right, with CZT OSEM reconstruction providing the highest definition of anatomical structures. eCAM = Brand name for single-photon emission computed tomography camera; FBP = Filtered back projection; CZT = Cadmium–zinc–telluride; OSEM = Ordered-subset expectation maximization.

Recent advances, especially in solid-state cadmium–zinc–telluride (CZT) cameras and pinhole collimators, have allowed for resolutions of up to 3-6 mm [2] plus higher white/gray matter contrast [3]. This should translate to increases in diagnostic accuracy, similar to trends reported in other fields [4,5], but head-to-head comparisons of CZT SPECT vs. SPECT/CT (using filtered back projection [FBP] or ordered-subset expectation maximization [OSEM]) with regard to neurodegenerative diseases are currently scarce (Figure 1). Also of concern is the training regimen required to adapt to CZT; low availability and higher costs to train may result in readers being unfamiliar with the higher resolution and contrast.

Currently, studies have shown that amyloid positron emission tomography (PET) and tau PET have promise for early detection of Alzheimer’s disease (AD) [6,7], but their use in clinical practice is limited. In this regard, cerebral blood flow SPECT and [^18^F] Fluorodeoxyglucose (FDG) PET remain useful for AD diagnoses. In Japan, cerebral blood flow SPECT is covered by insurance for the clinical diagnosis of AD, and increased resolution from CZT technology is expected to directly translate into improved diagnostic accuracy. However, direct comparisons of CZT and eCAM SPECT differences in improved spatial resolution on interrater diagnostic accuracy in AD diagnosis are scarce in the literature.

With the current shortcomings in diagnostic accuracy, AD serves as an excellent challenge for the improved resolution offered by CZT. Therefore, we conducted this study based on the research question: Does inter-reader reliability and diagnostic accuracy improve as spatial resolution increases? To test the utility of CZT SPECT as a useful diagnostic intervention versus typical SPECT/CT, we conducted a clinical trial to distinguish between patients with AD (known for patterns of decreased cerebral blood flow) and other patients.

## 2. Materials and Methods

### 2.1. Participants

This single-group, open-label, uncontrolled, diagnostic, objective, exploratory clinical trial enrolled patients with cerebrovascular disorders or neurodegenerative diseases who underwent SPECT to evaluate cerebral blood flow at the University of Tsukuba Hospital between 1 November 2019 and 31 March 2022. Inclusion criteria were consent to undergo additional imaging using CZT SPECT after conventional eCAM SPECT and an age of 20 years or older at the time of consent. Exclusion criteria were an inability to keep the head still for 20 min, acute cases (i.e., injuries or sudden afflictions such as hematomas), pregnancy, or being deemed unsuitable by the principal or sub-investigators. After exclusion, 29 patients were finally enrolled.

### 2.2. Ethics

This study was approved by the Institutional Review Board of the University of Tsukuba (Approval #TCRB19-009), and written informed consent was obtained from individual participants. This study complied with the tenets of the latest revision (2013) of the Declaration of Helsinki. This trial was registered in the Japan Registry of Clinical Trials (jRCT1032190116).

### 2.3. SPECT Scanning

An intravenous injection of approximately 600 MBq (16.2 mCi) of 99mTc-ECD was administered with the patient lying in a supine position with eyes closed in a dimly lit, quiet room. Ten minutes after the injection of 99mTc-ECD, the first SPECT scan was performed using a traditional, Anger-type, dual-headed γ-camera (ECAM, Siemens, Munich, Germany) equipped with a NaI detector and high-resolution fan beam collimators. For each detector, projection data were obtained in a 128 × 128 format for 45 angles at 20 s per angle. A Butterworth filter was used for image reconstruction at 0.5 cycle/cm, with attenuation correction using the Chang method.

Immediately after the first scan, the second scan was performed using a dual-head cadmium–zinc–telluride (CZT) SPECT/CT camera (Discovery NM 670CZT, GE Healthcare, Milwaukee, WI, USA). For each detector, projection data were obtained in a 128 × 128 format for 45 angles at 20 s per angle. A Butterworth filter was used for image reconstruction at 0.5 cycle/cm, with attenuation correction using CT. For eCAM data, reconstruction was performed using the filtered back projection (FBP) method with Chang attenuation correction, as our eCAM system is a standalone SPECT without CT capability. While OSEM reconstruction is technically available for this system, FBP with the Chang method is standard for brain perfusion SPECT at our institution due to optimal Chang correction and FBP algorithm compatibility. For CZT data, which benefit from integrated CT-based attenuation correction, reconstruction was performed using both FBP and the ordered subset expectation maximization (OSEM) method. For OSEM reconstruction of CZT data, we used 2 iterations with 10 subsets, reflecting the standard clinical protocol for brain perfusion imaging with the GE Discovery NM 670CZT system. This parameter selection balances resolution recovery with noise amplification, as additional iterations would enhance resolution but also amplify noise. To compare gray/white matter contrast between systems on equivalent terms, we used data processed with FBP from both systems. For image evaluation by clinicians, we used CZT data processed with OSEM since CZT with OSEM contrast is superior to CZT with FBP, as reported [8], and represents the standard clinical protocol for this system. To ensure valid comparisons between the two systems, identical post-processing filter parameters were implemented. For both eCAM and CZT SPECT reconstructions, a Butterworth filter with a cutoff frequency of 0.5 cycles/cm was applied uniformly across all images.

### 2.4. Image Assessment of Gray/White Matter Contrast

All participants were evaluated via the previously reported gray matter (GM)/white matter (WM) contrast method [9]. Briefly, images were spatially normalized with SPM12 (https://www.fil.ion.ucl.ac.uk/spm/software/spm12/ accessed on 2 April 2025). Then, we extracted mean gray matter (GM) and white matter (WM) counts within regions of interest, as defined by a previous report [9], and the ratio of GM/WM counts was calculated. For regional uptake ratio calculations, we used predefined anatomical masks from the AAL atlas, specifically bilateral posterior cingulate gyri, precunei, hippocampi, inferior parietal lobules, superior temporal gyri, and cerebellum. These masks were applied to the spatially normalized images, and mean counts within each region were extracted using an in-house script. The cerebellar reference region was chosen as it is typically spared in early Alzheimer’s disease, providing a stable reference for normalizing regional uptake values. 

### 2.5. Image Evaluation by Trained Readers

Two experienced clinicians conducted the visual evaluation of SPECT images: one radiologist with 24 years of clinical experience, including 18 years as a certified nuclear medicine specialist, and one psychiatrist with 23 years of clinical experience and 15 years of experience in nuclear medicine image interpretation. Both readers had extensive experience with conventional SPECT imaging but limited prior exposure to CZT SPECT technology for brain imaging. This disparity in experience was deliberately maintained without pre-study training on CZT images, as a key research objective was to evaluate the impact of transitioning to new technology without specialized training—a scenario that reflects the current clinical paradigm, since CZT technology is relatively new in neuropsychiatric applications. Images were displayed using a standardized protocol consisting of approximately 20 axial slices of the whole brain presented in a rainbow color scale. Both readers evaluated all images independently, without access to clinical information or prior imaging results, to minimize bias. Both readers had extensive experience with conventional SPECT imaging but limited prior exposure to CZT SPECT technology for brain imaging; this reflects the current clinical paradigm since this technology is relatively new in neuropsychiatric applications.

As Alzheimer’s disease is known to have a specific cerebral blood flow pattern (e.g., a decreased posterior cingulate, parietal, or hippocampus), two clinicians (readers) were trained to identify these specific patterns. However, there is no quantitative measurement possible, so the following visual scoring assessment was used:

0: No abnormality;
1: Pathology other than AD;
2: Possibly AD;
3: High possibility of AD.

Image scores for each reader from the same patient but a different imaging system were then compared. It is important to note that the readers who evaluated the SPECT images in this study did not participate in determining the diagnostic standard for Alzheimer’s disease. The diagnosis of AD was established independently using DSM-5 criteria prior to and separate from the SPECT evaluation process. This separation ensured an unbiased assessment of the imaging techniques’ diagnostic utility.

### 2.6. Statistical Analysis

Statistical analyses were performed using JASP (Version 0.18.3) software and custom analysis scripts. For comparing gray matter/white matter contrast between eCAM and CZT SPECT, paired t-testing was used as the Shapiro–Wilk test confirmed normal distribution (*p* = 0.944). For comparing diagnostic performance metrics (sensitivity, specificity, accuracy, positive predictive value [PPV], negative predictive value [NPV]) between the two imaging modalities, we initially considered McNemar’s test. However, due to the small sample size, particularly the limited number of AD cases (*n* = 8), conventional statistical testing could not provide reliable *p*-values. Therefore, we employed bootstrap resampling to generate more robust statistical inferences. We performed 10,000 bootstrap iterations, resampling with replacement while maintaining the same proportion of AD (*n* = 8) and non-AD (*n* = 21) cases. For each iteration, we calculated sensitivity, specificity, accuracy, PPV, and NPV for both imaging modalities and both readers. This approach allowed us to generate empirical 95% confidence intervals for each diagnostic metric and calculate empirical *p*-values for the differences observed between modalities.

To assess inter-reader agreement for binary classification (AD vs. non-AD), Cohen’s kappa statistic was calculated using bootstrap resampling. Ratings of 0–1 were classified as non-AD, and ratings of 2–3 were classified as AD suspicion for the binary analysis. A *p*-value of less than 0.05 was considered statistically significant.

To address image features beyond gray/white matter contrast, we performed additional quantitative analyses of regional cerebral blood flow patterns using the Automated Anatomical Labeling (AAL) atlas [10]. For each patient, we calculated several key imaging metrics: coefficient of variation (CoV), regional uptake ratios relative to the cerebellum, and signal-to-noise ratio (SNR). Regional uptake ratios were calculated by dividing the mean counts in specific brain regions (posterior cingulate, precuneus, hippocampus, parietal inferior, and temporal superior) by the mean counts in the cerebellar reference region (Cerebellum_6_R). SNR was estimated by dividing the mean cortical signal by the standard deviation of subcortical regions. Paired t-tests were used to compare these metrics between eCAM and CZT SPECT images, with a significance level of *p* < 0.05.

## 3. Results

### 3.1. Participant Characteristics

A total of 29 participants were enrolled in the study, with a mean age of 60.9 ± 17.6 years (range: 26–86 years), and 69% (*n* = 20) were female. Among these, 8 patients (27.6%) met the DSM-5 criteria [11] for Alzheimer’s disease, while 21 patients (72.4%) had other neurodegenerative or psychiatric disorders. Table 1 summarizes the demographic and clinical characteristics of the AD and non-AD subgroups. The AD group (*n* = 8) had a mean age of 72.1 ± 11.1 years (range: 52–84 years), was predominantly female (75%, *n* = 6), and had a mean Mini-Mental State Examination (MMSE) score of 19.6 ± 3.9. The diagnoses in this group included pure AD and AD with comorbid cerebrovascular disease. The non-AD group (*n* = 21) was significantly younger, with a mean age of 56.6 ± 17.9 years (range: 26–83 years), and was 66.7% (*n* = 14) female. This group included patients with a diverse range of conditions, including mild cognitive impairment, mood disorders (depression, bipolar disorder), frontotemporal lobar degeneration, Lewy body disease, vascular dementia, and other neurological conditions (e.g., post-NMDA receptor encephalitis). The mean MMSE score in the non-AD group, when available, was 26.4 ± 3.7. The significant age difference between groups (*p* < 0.05) represents a potential confounder, as the AD group was approximately 15.5 years older than the non-AD group, consistent with the known epidemiology of these conditions. All patients completed the study, and no adverse events were observed.

### 3.2. Gray Matter/White Matter Contrast Comparison Between eCAM and CZT SPECT

The contrast was 1.615 ± 0.096 for CZT and 1.458 ± 0.068 for eCAM, with CZT being significantly higher (*p* < 0.001, Cohen’s d = 0.38). Figure 1 summarizes the differences among all utilized imaging methods (eCAM with FBP, CZT with FBP, and CZT with OSEM). When comparing diagnostic performance metrics between eCAM and CZT SPECT, both readers demonstrated similar patterns (Table 2). For the psychiatrist, sensitivity decreased from 75.0% (6/8) with eCAM to 62.5% (5/8) with CZT, while specificity improved slightly from 66.7% (14/21) to 71.4% (15/21). Overall accuracy remained unchanged at 69.0% (20/29). For the radiologist, sensitivity was maintained at 87.5% (7/8) for both modalities, but specificity decreased from 57.1% (12/21) with eCAM to 42.9% (9/21) with CZT, resulting in a reduction in overall accuracy from 65.5% (19/29) to 55.2% (16/29). Due to the small sample size, formal statistical comparison using McNemar’s test could not be reliably performed.

Inter-reader agreement for binary classification (AD vs. non-AD) was assessed using Cohen’s kappa. For eCAM SPECT, the observed agreement between readers was 62.1% with a kappa of 0.249, indicating fair agreement. For CZT SPECT, the observed agreement decreased substantially to 37.9% with a negative kappa of −0.155, indicating poor agreement that was worse than would be expected by chance alone. This significant reduction in inter-reader concordance with CZT SPECT, despite its improved image contrast, suggests that unfamiliarity with the higher resolution images negatively impacted diagnostic agreement between readers.

Quantitative analysis of image features beyond gray/white matter contrast revealed significant differences between eCAM and CZT SPECT. The CoV was significantly higher for CZT (15.01%) compared to eCAM (13.71%; *p* < 0.001), indicating greater regional variability in the CZT images. Conversely, SNR was significantly lower for CZT (4.79) than for eCAM (5.28; *p* < 0.001), suggesting potentially higher noise levels in CZT images despite their improved spatial resolution.

Regional uptake ratios relative to the cerebellum showed varying differences between the two imaging systems. The posterior cingulate/cerebellar ratio was significantly lower in CZT images (0.657) compared to eCAM (0.721), representing an 8.9% reduction (*p* < 0.001). This finding is noteworthy as posterior cingulate hypoperfusion is a key diagnostic marker for Alzheimer’s disease. In contrast, parietal/cerebellar and temporal/cerebellar ratios showed minimal differences between modalities (0.5% and 0.9% differences, respectively; *p* > 0.05).

### 3.3. Score Differences Between Readers with Regard to eCAM vs. CZT SPECT

Table 3 summarizes the differences in diagnostic performance between eCAM and CZT SPECT for both readers. Bootstrap analysis (10,000 iterations) provided the following 95% confidence intervals (95% CI) for the psychiatrist: sensitivity of 0.50–1.00 for eCAM and 0.38–0.88 for CZT; specificity of 0.52-0.81 for both eCAM and CZT. For the radiologist, the 95% CIs were sensitivity of 0.63-1.00 for both eCAM and CZT; specificity of 0.43–0.71 for eCAM and 0.24–0.62 for CZT.

The bootstrap analysis revealed a considerable overlap in the confidence intervals for all diagnostic metrics between eCAM and CZT for both readers. The empirical *p*-values for differences in accuracy were *p* = 0.84 for the psychiatrist (eCAM vs. CZT) and *p* = 0.27 for the radiologist. While the radiologist’s specificity showed the largest numerical decrease from eCAM (0.571) to CZT (0.429), the bootstrap analysis yielded *p* = 0.13, indicating that this difference did not reach statistical significance at the conventional threshold.

The bootstrap analysis of inter-reader agreement yielded a 95% confidence interval for Cohen’s kappa of 0.05–0.45 for eCAM and −0.35–0.05 for CZT, confirming that the observed decrease in agreement (from fair to poor) between readers when using CZT SPECT was statistically significant (*p* = 0.02).

## 4. Discussion

Constant refinement of clinical guidelines and advances in imaging have attempted to simplify the diagnosis of Alzheimer’s disease, but the multi-factorial pathology remains a challenging barrier. In the current report, cerebral blood flow images obtained with the latest semiconductor detector system showed a statistically significant improvement in gray matter/white matter contrast compared to the conventional model. On the other hand, the positive diagnosis rate was slightly lower for qualitative diagnosis using images, suggesting that unfamiliarity with higher resolution imaging must be countered with training.

While amyloid and tau PET imaging offer higher specificity advantages for AD pathology, cerebral perfusion SPECT provides several practical advantages, including lower cost, wider accessibility (especially when cyclotron facilities are unavailable), and insurance coverage in multiple countries. Although SPECT cannot directly visualize amyloid or tau deposits, detection of functional changes in cerebral blood flow patterns may identify neurodegenerative or cerebrovascular disorders [12,13] earlier than structural imaging. This information is complementary to molecular imaging techniques when used with proper diagnostic algorithms and can aid in diagnosis. CZT SPECT technology, with its improved resolution and significantly higher gray/white matter contrast, should be superior to conventional SPECT in diagnostic capability.

The most commonly reported SPECT systems, such as our eCAM system, are Anger-type systems based on the use of NaI (sodium iodide) as the scintillator with an adjacent vacuum tube called a photomultiplier tube. The cylindrical shape of the vacuum tube creates a significant limitation as it cannot cover the entire area of the scintillator, resulting in signal loss and irreversible degradation of image quality. A SPECT system using cadmium–zinc–telluride (CZT) as a scintillator and photomultiplier has recently become commercially available. CZT exploits the ability of a semiconductor to convert incident radiation into an electrical signal at room temperature within the semiconductor, removing the need for a vacuum tube and reducing signal loss [14]. Additionally, radiation is detected by a charged screen with smaller holes than standard pixels, in which 5 eV is sufficient to generate an electron–hole pairing; this results in higher resolution, contrast, and sensitivity [14] (Figure 1). Initially, CZT devices were developed and dedicated to cardiac use, but a whole-body device has recently become commercially available and was installed at our hospital in 2018. However, due to high cost, CZT systems have not seen widespread use, either domestically or internationally, and few studies report using this device. To the best of our knowledge, only reports on the cardiac sympathetic nerve [15], brain dopamine receptors [16], and bone scintigraphy [17] have been published using CZT detector technology.

Conventional imaging cannot match the gray matter/white matter contrast of next-generation CZT SPECT, but enhanced imaging capability may not increase diagnostic accuracy from current “good enough” imaging with regard to blood flow. To highlight any differences in SPECT findings between the two systems, we observed that, while CZT SPECT showed significantly higher gray/white matter contrast (1.615 ± 0.096 vs. 1.458 ± 0.068, *p* < 0.001), the actual pattern of cerebral blood flow abnormalities remained consistent, regardless of technology. In patients diagnosed with Alzheimer’s disease, both systems revealed characteristic bilateral temporoparietal hypoperfusion and posterior cingulate hypoperfusion, albeit with sharper boundaries on CZT images. While it is true that CZT resolution was useful in discrimination and visualization of anatomical structures, especially smaller areas, this did not translate to notable differences in disease patterns. This indicates that the diagnosis of complex neurodegenerative pathologies relies on more than gray/white matter contrast. 

Our comprehensive analysis of image features beyond gray/white matter contrast provides insights into why improved resolution does not linearly translate to enhanced diagnostic accuracy. CZT SPECT, in spite of the marked contrast advantage, also demonstrated higher regional variability (CoV) and lower signal-to-noise ratio compared to conventional eCAM SPECT. Such characteristics may introduce patterns that result in misinterpretation through unfamiliarity. Notably, the reduced posterior cingulate/cerebellar ratio in CZT images compared to eCAM may affect the assessment of hypoperfusion patterns typical of Alzheimer’s disease. Despite advanced resolution, subtle posterior cingulate hypoperfusion may thus escape notice when insufficiently trained readers evaluate CZT images.

The higher coefficient of variation (CoV) and lower signal-to-noise ratio (SNR) we observed in CZT images likely complicated diagnostic accuracy. Increased regional heterogeneity (higher CoV) may increase the difficulty for readers to distinguish between normal physiological variability and disease-related hypoperfusion, particularly in borderline cases. Similarly, the reduced SNR may hide subtle perfusion changes that are critical for early AD detection. Readers accustomed to the smoother appearance and lower regional variability of conventional SPECT may misinterpret the more heterogeneous appearance of CZT images as pathological findings, potentially explaining some of the CZT false positives. These technical characteristics underscore that the simple transference of conventional SPECT interpretation skills to CZT imaging is insufficient.

The technical differences we observed between eCAM and CZT SPECT, particularly the inherently lower SNR and higher CoV in CZT images, warrant careful consideration. Alternative reconstruction approaches with more iterations or different filter parameters might potentially improve SNR at the cost of resolution, but would deviate from standard clinical protocols. Future studies must systematically optimize reconstruction parameters specifically for neurodegenerative disease assessment to find better balance points between resolution and noise characteristics for diagnostic purposes.

Our analysis revealed a significant reduction in the posterior cingulate/cerebellar ratio in CZT images compared to eCAM (−8.9%, *p* < 0.001). Given that posterior cingulate hypoperfusion is a key diagnostic marker for Alzheimer’s disease, this finding has important clinical implications. This altered ratio probably reflects technical rather than biological differences, as the same patients were imaged with both modalities in close succession. The superior spatial resolution of CZT detectors (3–6 mm vs. 10 mm) reduces partial volume effects differently across brain regions, particularly affecting smaller structures (e.g., the posterior cingulate). Additionally, the different attenuation correction methods (CT-based for CZT vs. Chang method for eCAM) and any inherent detector characteristics may contribute to these regional quantitative differences.

This finding highlights that established disease detection quantitative thresholds developed with conventional SPECT cannot be directly applied to CZT imaging. Technology-specific, regional ratio reference ranges must be generated, and readers should be trained to recognize that hypoperfusion patterns may present differently on CZT images. Our collective findings suggest that, while CZT SPECT offers superior image quality, the significant differences in image appearance may initially hinder diagnostic performance when readers trained on conventional SPECT encounter CZT images.

These observations enforce the requirements for specific training and time-on-instrument to become familiar with improved anatomical detail provided by CZT technology, as well as the modified quantitative relationships between key brain regions relevant to the diagnosis of neurodegenerative diseases. We emphasize the critical need for standardized training programs specifically designed for CZT SPECT interpretation in neurodegenerative diseases. Similar to how the easy Z-score imaging system (eZIS) standardized conventional SPECT interpretation, we propose the development of a CZT-specific image library and analytical framework for Alzheimer’s disease. The development of normal CZT databases and adjusted thresholds for statistical analysis would also be beneficial for optimal clinical implementation of this advanced technology. Given the significant investment in CZT technology, complementary investment in specialized training is essential to fully realize its clinical potential.

The cooperation of radiologists and psychiatrists in evaluating images for neurodegenerative diseases is crucial since radiologists can evaluate the overall blood flow from imaging scans, but psychiatrists use SPECT as a biomarker to arrive at diagnoses of cognitive diseases in concert with other specialized guidelines that undergo regular revision [18]. In our study, both the radiologist and psychiatrist experienced drops in all scoring parameters with CZT, indicating that training on AD-specific datasets would be useful for all members of an AD diagnosis team. Training methodologies with regard to SPECT exist regarding patient orientation, contrast agents, machine parameters, and image interpretation (https://www.asnc.org/fit_spect accessed on 2 April 2025). New developments in CZT SPECT for cardiology and other organ systems, such as attenuation correction with deep learning, must also be assimilated by both neuropsychiatrists and radiologists to assist in imaging cerebral pathologies [19,20]. In light of our results, experienced nuclear medicine physicians may need specialized, cooperative training in differences between Anger-type and CZT detectors with regard to final images, transformatory algorithms, artifacts unique to both types, and peculiarities in blood flow. In our study, the inter-reader concordance was significantly reduced with CZT SPECT despite its improved image contrast. Such a decrease in inter-reader agreement between eCAM and CZT SPECT must be interpreted cautiously, given our methodological limitations. With only two readers, we cannot definitively rule out that this finding reflects individual interpretative tendencies rather than a generalizable effect. However, both readers were experienced specialists with extensive conventional SPECT experience, suggesting that the observed disagreement likely stems at least partly from unfamiliarity with CZT-specific image characteristics.

Based on our findings, we propose the following practical framework for integrating CZT SPECT into existing diagnostic workflows:

1. Preparation Phase: Establish a multidisciplinary committee (radiologists, nuclear medicine specialists, psychiatrists, neurologists) to develop institution-specific protocols and create a comparative database of paired conventional and CZT images for training.
2. Transition Phase: Implement a 3-month overlap period using both technologies on selected patients, with structured joint reading sessions between radiologists and psychiatrists to identify and resolve interpretation discrepancies.
3. Implementation Phase: Maintain a double-reading workflow where both specialists independently evaluate each scan before reaching consensus, mitigating the reduced inter-reader agreement observed in our study.
4. Optimization Phase: Incorporate CZT-specific quantitative analysis tools and establish a feedback loop between clinical outcomes and imaging findings for continuous improvement. This framework acknowledges the complementary expertise of radiologists (image quality assessment) and psychiatrists (clinical integration) while formalizing their collaboration to minimize diagnostic divergence and maximize the benefits of CZT technology.

The most notable finding in our study was the significant decrease in inter-reader agreement when using CZT SPECT, dropping from fair agreement with eCAM (κ = 0.249) to poor agreement worse than chance (κ = −0.113, *p* < 0.001). This striking disagreement likely stems from three key factors: (1) the improved resolution of CZT images revealed features not visible on conventional SPECT, leading to divergent interpretations without standardized guidelines; (2) altered quantitative relationships, particularly the reduced posterior cingulate/cerebellar ratio (−8.9%), affected interpretation of this key AD diagnostic marker; and (3) higher regional variability and lower SNR introduced unfamiliar patterns that were inconsistently interpreted. These findings indicate that institutions adopting CZT technology should anticipate an initial period of reduced diagnostic concordance, necessitating structured training programs and CZT-specific databases before clinical deployment.

This study design was limited by a small sample size and a single-center location. Multi-center collaborations would enable larger cohorts and allow stratification of non-AD groups into specific disease categories for differential diagnostic analysis. Longitudinal studies evaluating the same readers’ performance over time would quantify learning curves and determine optimal training requirements. Such studies should incorporate structured training programs with progressive exposure to CZT cases of varying complexity; comprehensive quantitative diagnostic metrics, including ROC analysis; systematic reporting of full reconstruction parameters; and crossover reader-training experiments to test performance improvements as readers adapt to CZT-specific image characteristics. Tracking changes in sensitivity, specificity, and inter-reader agreement over time would establish evidence-based implementation guidelines, shortening the adaptation period and accelerating clinical benefits from CZT technology.

This study must acknowledge some additional limitations. A methodological consideration in our study design was the sequential acquisition protocol, where eCAM SPECT was always performed first, followed by CZT SPECT. This approach could have introduced order-related biases, namely patient fatigue during the second scan, which might have led to increased motion artifacts, while tracer redistribution over time may have altered the apparent distribution of cerebral blood flow. Although we minimized the interval between scans, the approximately 30 min delay before CZT imaging might have affected tracer distribution, particularly in regions with rapid kinetics. This sequential bias, if present, would most likely disadvantage CZT SPECT, potentially exaggerating the observed differences. Future studies should consider randomizing the imaging order or, if possible, use split-dose techniques to minimize these temporal effects. Additionally, sample sizes were small (n = 29, with only eight AD cases) due to the study period overlapping with the COVID-19 pandemic, which severely restricted our recruitment capabilities. This limited sample size reduced statistical power and prevented robust subgroup analyses. The imbalance between AD and non-AD cases further constrained the reliability of our sensitivity and specificity calculations. To address these limitations, we propose future multi-center studies with larger, more balanced cohorts of AD and non-AD cases. Such collaborative efforts would provide greater statistical power, enable meaningful subgroup analyses, and improve the generalizability of results across diverse clinical settings. With the relaxation of pandemic measures, we anticipate that future planned studies will successfully recruit more patients, allowing for more definitive conclusions about the diagnostic utility of CZT SPECT technology in neurodegenerative diseases. We also acknowledge that our “non-AD” group includes a heterogeneous mix of conditions that could potentially confound specificity assessments. Furthermore, in this study, we compared Alzheimer’s disease (AD) patients to patients with other neurodegenerative diseases; however, sample size issues precluded any subgroup analyses of cerebrovascular disorder (CVD) patients. As CVD-specific cerebral blood flow patterns markedly differ from those of AD patients, any effect of high-resolution CZT SPECT imaging on discrimination between AD and CVD diagnoses remains reserved for future research. Such future studies must include comparative analyses spanning multiple disease categories, including CVD patients. The decrease in inter-reader agreement observed with CZT SPECT must be interpreted cautiously, given our methodological limitations. With only two readers, we cannot definitively rule out that this finding reflects individual tendencies rather than a generalizable effect. However, both readers were experienced specialists with extensive conventional SPECT experience, suggesting that unfamiliarity with CZT-specific tendencies was at least partly responsible. Additionally, our current study design precluded correlation analyses between quantitative image metrics (SNR, CoV, contrast) and diagnostic performance. Such analyses would require individual patient-level image quality metrics rather than the cohort-level metrics we calculated. In future studies with larger sample sizes, we plan to incorporate these individual metrics and examine how they correlate with diagnostic accuracy through scatter plots and regression analyses. This would provide valuable insights into which technical aspects of the imaging process have the greatest impact on clinical utility and could help identify specific technical thresholds that optimize diagnostic accuracy. A critical limitation is the potential for individual reader bias to significantly influence our findings. With only two readers evaluating all images, their specific interpretive tendencies may have disproportionately affected results, particularly the decrease in inter-reader agreement with CZT SPECT. Future studies should implement more robust diagnostic evaluation methods, including multiple readers with varying experience levels; standardized interpretation criteria specifically for CZT SPECT; multiple reading sessions with washout periods; and correlation with other biomarkers to establish ground truth beyond clinical diagnosis. Additionally, objective quantitative evaluation methods less susceptible to reader bias should complement visual assessments to better distinguish technology-related effects from reader-dependent variables. In the future, multi-center studies will involve dozens of psychiatrists and radiologists, allowing for a comparison between control and training groups to test the effectiveness of programs designed to improve diagnostic accuracy. In spite of these limitations, the utility of CZT was shown in the current study to be limited by interpreter familiarity.

## 5. Conclusions

In conclusion, we found that sensitivity, specificity, and accuracy were slightly lower with the CZT detector versus the traditional NaI detector, even if resolution and contrast were better. We also found decreases in all image scoring results from eCAM to CZT in both the participating radiologist and psychiatrist, who were unfamiliar and untrained on the new system. Training with standardized CZT image sets, focused on the stages of progressive, neurodegenerative diseases, will increase accuracy and serve to establish SPECT as a reliable biomarker in the diagnostic process. We recommend developing a CZT-specific equivalent to established tools like eZIS, providing a statistical comparison to normative databases specifically developed for this technology. Future studies should evaluate the learning curve across larger reader panels with standardized training interventions and objective pre-/post-assessments. This would help establish whether the diagnostic challenges we observed represent a generalizable phenomenon requiring systematic training or merely reflect individual reader variability.

## Figures and Tables

**Table 1:** Demographic and clinical characteristics of study participants.

Characteristic	All Participants (*n* = 29)	AD Group (*n* = 8)	Non-AD Group (*n* = 21)	*p*-Value
**Age (years)**				
Mean ± SD	60.9 ± 17.6	72.1 ± 11.0	56.6 ± 17.9	<0.05 *
Range	26–86	52–86	26–83	
**Sex, *n* (%)**				0.66
Female	20 (69.0)	6 (75.0)	14 (66.7)	
Male	9 (31.0)	2 (25.0)	7 (33.3)	
**MMSE ^†^**				
Mean ± SD	24.3 ± 4.9	19.8 ± 4.3	26.4 ± 3.7	<0.01 *
Range	15–30	15–27	20–30	
**Group, *n* (%)**				
Alzheimer’s disease (AD)	8 (27.6)	8 (100)	-	
Non-AD conditions ^‡^	21 (72.4)	-	21 (100)	

^†^ MMSE scores were available for all AD patients and 18 of 21 non-AD patients. ^‡^ Includes various neurodegenerative conditions (mild cognitive impairment, vascular dementia, frontotemporal lobar degeneration, Lewy body disease) and other neuropsychiatric disorders. * Statistically significant difference (*p* < 0.05).

**Table 2:** Comparison of quantitative image features between eCAM and CZT SPECT.

Image Feature	eCAM SPECT	CZT SPECT	Difference (%)	*p*-Value
Gray/White Matter Contrast	1.458 ± 0.068	1.615 ± 0.096	+10.8%	<0.001
Coefficient of Variation (CoV)	13.71%	15.01%	+9.5%	<0.001
Signal-to-Noise Ratio (SNR)	5.28	4.79	−9.5%	<0.001
Posterior Cingulate/Cerebellar Ratio	0.721	0.657	−8.9%	<0.001
Parietal/Cerebellar Ratio	0.77	0.774	+0.5%	0.652
Temporal/Cerebellar Ratio	0.862	0.87	+0.9%	0.413
Hippocampus/Cerebellar Ratio	0.746	0.728	−2.4%	0.047

**Table 3:** Diagnostic performance metrics of eCAM and CZT SPECT with bootstrap 95% confidence intervals.

Metric	Psychiatrist		Radiologist	
Sensitivity	0.750 (0.375–1.000)	0.625 (0.250–0.875)	0.875 (0.625–1.000)	0.875 (0.625–1.000)
Specificity	0.667 (0.476–0.857)	0.667 (0.476–0.857)	0.571 (0.333–0.762)	0.429 (0.238–0.667)
PPV	0.462 (0.286–0.700)	0.417 (0.222–0.667)	0.438 (0.312–0.615)	0.368 (0.267–0.500)
NPV	0.875 (0.733–1.000)	0.824 (0.688–0.947)	0.923 (0.778–1.000)	0.900 (0.714–1.000)
Accuracy	0.690 (0.517–0.862)	0.655 (0.483–0.828)	0.655 (0.483–0.828)	0.552 (0.379–0.724)

Values represent point estimates with bootstrap 95% confidence intervals in parentheses (10,000 iterations). PPV = Positive predictive value; NPV = Negative predictive value. No statistically significant differences were found between eCAM and CZT for any metrics (all *p* > 0.05).

## Data Availability

All data obtained are presented and available in the manuscript.

## References

[1] Matsuda H., Mizumura S., Nagao T., Ota T., Iizuka T., Nemoto K., Takemura N., Arai H., Homma A. (2007). Automated Discrimination between Very Early Alzheimer Disease and Controls Using an Easy Z-Score Imaging System for Multicenter Brain Perfusion Single-Photon Emission Tomography. AJNR Am. J. Neuroradiol..

[2] Khalil M.M., Tremoleda J.L., Bayomy T.B., Gsell W. (2011). Molecular SPECT Imaging: An Overview. Int. J. Mol. Imaging.

[3] Bordonne M., Chawki M.B., Marie P.-Y., Zaragori T., Roch V., Grignon R., Imbert L., Verger A. (2020). High-Quality Brain Perfusion SPECT Images May Be Achieved with a High-Speed Recording Using 360° CZT Camera. EJNMMI Phys..

[4] Eskreis-Winkler S., Sung J.S., Dixon L., Monga N., Jindal R., Simmons A., Thakur S., Sevilimedu V., Sutton E., Comstock C. (2023). High-Temporal/High-Spatial Resolution Breast Magnetic Resonance Imaging Improves Diagnostic Accuracy Compared With Standard Breast Magnetic Resonance Imaging in Patients With High Background Parenchymal Enhancement. J. Clin. Oncol..

[5] Hagar M.T., Soschynski M., Saffar R., Rau A., Taron J., Weiss J., Stein T., Faby S., von Zur Muehlen C., Ruile P. (2023). Accuracy of Ultrahigh-Resolution Photon-Counting CT for Detecting Coronary Artery Disease in a High-Risk Population. Radiology.

[6] Hameed S., Fuh J.-L., Senanarong V., Ebenezer E.G.M., Looi I., Dominguez J.C., Park K.W., Karanam A.K., Simon O. (2020). Role of Fluid Biomarkers and PET Imaging in Early Diagnosis and Its Clinical Implication in the Management of Alzheimer’s Disease. J. Alzheimers Dis. Rep..

[7] Tagai K., Ono M., Kubota M., Kitamura S., Takahata K., Seki C., Takado Y., Shinotoh H., Sano Y., Yamamoto Y. (2021). High-Contrast In Vivo Imaging of Tau Pathologies in Alzheimer’s and Non-Alzheimer’s Disease Tauopathies. Neuron.

[8] Wu W., Zhang R., Zhou Y., Wang S., Shen Y., Li N., Tan J., Zheng W., Jia Q., Meng Z. (2023). Impacts of Different Reconstruction Methods on the Image Quality of Cadmium-Zinc-Telluride-Based Single Photon Emission Computed Tomography/computed Tomography Pulmonary Perfusion Imaging. Nucl. Med. Commun..

[9] Ikari Y., Akamatsu G., Nishio T., Ishii K., Ito K., Iwatsubo T., Senda M. (2016). Phantom Criteria for Qualification of Brain FDG and Amyloid PET across Different Cameras. EJNMMI Phys..

[10] Tzourio-Mazoyer N., Landeau B., Papathanassiou D., Crivello F., Etard O., Delcroix N., Mazoyer B., Joliot M. (2002). Automated Anatomical Labeling of Activations in SPM Using a Macroscopic Anatomical Parcellation of the MNI MRI Single-Subject Brain. Neuroimage.

[11] American Psychiatric Association, American Psychiatric Association (2013). DSM-5 Task Force. Diagnostic and Statistical Manual of Mental Disorders: DSM-5.

[12] Oku N., Kashiwagi T., Hatazawa J. (2010). Nuclear Neuroimaging in Acute and Subacute Ischemic Stroke. Ann. Nucl. Med..

[13] Austin B.P., Nair V.A., Meier T.B., Xu G., Rowley H.A., Carlsson C.M., Johnson S.C., Prabhakaran V. (2011). Effects of Hypoperfusion in Alzheimer’s Disease. J. Alzheimers Dis..

[14] Ito T., Matsusaka Y., Onoguchi M., Ichikawa H., Okuda K., Shibutani T., Shishido M., Sato K. (2021). Experimental Evaluation of the GE NM/CT 870 CZT Clinical SPECT System Equipped with WEHR and MEHRS Collimator. J. Appl. Clin. Med. Phys..

[15] Okano N., Osawa I., Tsuchihashi S., Takahashi M., Niitsu M., Matsunari I. (2019). High-Speed Scanning of Planar Images Showing 123I-MIBG Uptake Using a Whole-Body CZT Camera: A Phantom and Clinical Study. EJNMMI Res..

[16] Bani Sadr A., Testart N., Tylski P., Scheiber C. (2019). Reduced Scan Time in 123I-FP-CIT SPECT Imaging Using a Large-Field Cadmium-Zinc-Telluride Camera. Clin. Nucl. Med..

[17] Yamane T., Kondo A., Takahashi M., Miyazaki Y., Ehara T., Koga K., Kuji I., Matsunari I. (2019). Ultrafast Bone Scintigraphy Scan for Detecting Bone Metastasis Using a CZT Whole-Body Gamma Camera. Eur. J. Nucl. Med. Mol. Imaging.

[18] Tahami Monfared A.A., Phan N.T.N., Pearson I., Mauskopf J., Cho M., Zhang Q., Hampel H. (2023). A Systematic Review of Clinical Practice Guidelines for Alzheimer’s Disease and Strategies for Future Advancements. Neurol. Ther..

[19] Zavadovsky K.V., Mochula A.V., Maltseva A.N., Shipulin V.V., Sazonova S.I., Gulya M.O., Liga R., Gimelli A. (2022). The Current Status of CZT SPECT Myocardial Blood Flow and Reserve Assessment: Tips and Tricks. J. Nucl. Cardiol..

[20] Ochoa-Figueroa M., Valera-Soria C., Pagonis C., Ressner M., Norberg P., Sanchez-Rodriguez V., Frias-Rose J., Good E., Davidsson A. (2024). Diagnostic Performance of a Novel Deep Learning Attenuation Correction Software for MPI Using a Cardio Dedicated CZT Camera. Experience in the Clinical Practice. Rev. Esp. Med. Nucl. Imagen Mol..

